# Modelling the Potential Geographic Distribution of Two *Trissolcus* Species for the Brown Marmorated Stink Bug, *Halyomorpha halys*

**DOI:** 10.3390/insects12060491

**Published:** 2021-05-25

**Authors:** Tania Yonow, Darren J. Kriticos, Noboru Ota, Gonzalo A. Avila, Kim A. Hoelmer, Huayan Chen, Valerie Caron

**Affiliations:** 1 CSIRO, Health and Biosecurity, GPO Box 1700, Canberra, ACT 2600, Australia; noboru.ota@csiro.au; 2 The New Zealand Institute for Plant and Food Research Limited, Private Bag 92169, Auckland Mail Centre, Auckland 1142, New Zealand; gonzalo.avila@plantandfood.co.nz; 3 Beneficial Insect Introduction Research Unit, USDA, ARS, 501 South Chapel Street, Newark, DE 19713, USA; kim.hoelmer@usda.gov; 4 State Key Laboratory of Biocontrol, School of Life Sciences/School of Ecology, Sun Yat-sen University, Guangzhou 510275, China; chenhuayan@mail.sysu.edu.cn

**Keywords:** *Trissolcus mitsukurii*, *Trissolcus japonicus*, *Halyomorpha halys*, CLIMEX, niche modelling, potential distribution

## Abstract

**Simple Summary:**

The brown marmorated stink bug, *Halyomorpha halys* (Stål) (Hemiptera: Pentatomidae), native to Asia, has been accidentally introduced to Europe and North America, where it has become a key pest by feeding on numerous important crops. Although *H. halys* has not yet established in Australia, there is a general consensus that this is only a matter of time, and thus, it is prudent to investigate management options. Previous studies have modelled the potential distribution of *H. halys* and one of its principal natural enemies, *Trissolcus japonicus* (Ashmead) (Hymenoptera: Scelionidae). Here, we developed a similar model of the potential distribution of *Trissolcus mitsukurii* (Ashmead), which is a primary parasitoid of *H. halys* in Japan, and which was introduced to Australia in the 1960s to control another introduced pest. We used the three models to examine the overlap in the projected distributions of both *T. mitsukurii* and *T. japonicus* with *H. halys*, and to assess the potential for the two *Trissolcus* species to help mitigate the impacts of *H. halys* in its global adventive range.

**Abstract:**

The brown marmorated stink bug, *Halyomorpha halys* (Stål) (Hemiptera: Pentatomidae), is native to northeast Asia. It was accidentally introduced to Europe and North America, where it has become a key pest, feeding on many important crops. Previous eco-climatic niche modelling indicates that *H. halys* could expand its distribution vastly, and numerous border interceptions of this pest in many countries, including Australia and New Zealand, indicate that it would be prudent to prepare for its eventual arrival. Similar niche modelling was used to assess the potential distribution of *Trissolcus japonicus* (Ashmead) (Hymenoptera: Scelionidae), the key parasitoid of *H. halys* in China. *Trissolcus mitsukurii* (Ashmead) is one of the main parasitoids of *H. halys* in Japan. It is known to have existed in Australia since the early 20th century and was also specifically introduced to Australia in the 1960s, and it has now also invaded Italy. We used CLIMEX to model the climatic niche of *T. mitsukurii* to estimate its global potential distribution. We found that *T. mitsukurii* should be able to significantly expand its range globally, and that there is a significant degree of overlap in the projected ranges of *T. mitsukurii*, *T. japonicus* and *H. halys*. From a biological control perspective, this implies that the two *Trissolcus* species may be able to help mitigate the potential impacts of *H. halys*.

## 1. Introduction

The brown marmorated stink bug, *Halyomorpha halys* (Stål) (Hemiptera: Pentatomidae), is native to Asia (China, Japan, Korea and Taiwan) [1]. It was accidentally introduced to Europe and North America, where it has become a key pest, feeding on many important crops [2,3,4]. It is also considered to be a nuisance pest when it invades homes seeking overwintering sites [5,6]. Climatic modelling indicates the potential for *H. halys* to significantly expand its distribution in both North America and Europe, and to invade Central and South America, Africa, Australia and New Zealand [7]. Although *H. halys* has not yet established in New Zealand or Australia, there have been numerous border interceptions [7,8,9], and it may be only a matter of time before it becomes established in these countries. As *H. halys* is projected to establish in prime horticultural areas in both Australia and New Zealand [7], it is prudent to prepare for its arrival. Classical biological control is a potentially important option for managing *H. halys*, and two parasitoids have been identified as potential biological control agents against *H. halys*: *T. japonicus* (Ashmead) (Hymenoptera: Scelionidae) (=*T. halyomorphae* Yang) and *T. mitsukurii* (Ashmead) [10].

*Trissolcus japonicus* is a significant egg parasitoid of *H. halys* in China [11]. *Trissolcus japonicus* has apparently invaded North America [3,12,13,14] and is now found in 13 US states, the District of Columbia and two Canadian provinces [15,16]. In Europe, it recently invaded Italy [17] and Switzerland [18]. Of all the *Trissolcus* parasitoids associated with *H. halys* in China, *T. japonicus* has the highest parasitism rate and it is the most effective egg parasitoid recorded [1,11]. A potential risk with using this species as a biological control agent is that it is known to be oligophagous, and can parasitise and develop in eggs of other species in the families Pentatomidae and Scutelleridae [8,12,19,20,21]. Nonetheless, *T. japonicus* has been approved in Italy for biological control of *H. halys* [22], and New Zealand has recently conditionally approved the release of *T. japonicus* in the event that *H. halys* becomes established [8,23]. *Trissolcus japonicus* is clearly highly mobile, and has proven itself to be an effective invader in areas far from its native range [3,12,13,14,15,16,17,18]. Given that thousands of individuals of *H. halys* have been intercepted at the New Zealand border with interceptions growing exponentially [24], it seems likely that it will eventually become established in New Zealand, and hence, that *T. japonicus* will subsequently be released there. The demonstrated mobility of *T. japonicus*, the close proximity of New Zealand and Australia and the strong trade flows between these countries mean that it is likely to eventually also arrive in Australia, despite it not being considered as a potential biological control agent for *H. halys* due to potential risks to native pentatomids [10].

*Trissolcus mitsukurii* is considered a key parasitoid of *H. halys* in Japan [6], and is now present in Australia [25,26] and Europe [17,27]. In preparation for the likely future arrival of *H. halys* in Australia, it is worth considering *T. mitsukurii* as a potential biological control agent for *H. halys* in Australia [10]. For this study, we presume that the native distribution of *T. mitsukurii* is Asia, most likely China, Japan, South Korea, Taiwan, Thailand and Vietnam. We also presume that adventive populations reached Australia (1914), Papua (Indonesia) (1958) and Sri Lanka (1914), possibly as a hitch-hiker along trade routes. We presume it to be a naturalised exotic in Australia, because whilst we do not know if it is native to Australia or if it arrived in the early 20th century (intentionally or unintentionally introduced), we do know a population was introduced to Australia from Japan in 1962 to control *Nezara viridula* L. (Hemiptera: Pentatomidae), the green vegetable bug [25]. Apart from four records dated prior to the 1962 introduction (two of which were identified in 1914 as *Telenomus oecleoides*, now considered a synonym of *T. mitsukurii* [26]), all other location records in Australia are dated post-1975, in states and a territory where that 1962 introduction was released, suggesting that the 1962 release is perhaps the primary source of current populations. Population genetics should be able to resolve the issue of where Australian *T. mitsukurii* populations originated, but this also requires a much better knowledge of where the species occurs, given that there is very limited information available on its current distribution in Australia. Accurate knowledge of its distribution is important in the event of an introduction of *H. halys*, as it may become prudent to redistribute *T. mitsukurii* to new areas, and it is important to know whether it can establish in areas that may also support *H. halys* populations [7]. To assist in sampling for *T. mitsukurii* in Australia, it also helps to know which regions are potentially climatically suitable for establishment.

Climate is the primary determinant of a species’ distribution [28,29,30,31]. There have been countless studies in pest risk management looking to assess the suitability of novel areas for species invasion, all focusing on how the climatic conditions within the current distribution compare to the novel areas under consideration, whether these be under current [32,33,34] or future climate scenarios [35,36,37,38]. Following previous studies using CLIMEX to assess the potential distribution of *H. halys* [7] and *T. japonicus* [39], we used CLIMEX to assess the potential distribution of *T. mitsukurii*.

Very little is known about this species, and we initially thought that we did not have sufficient information for the more comprehensive Compare Locations analysis. Consequently, we commenced our investigation with a Match Climates (Regional) analysis (hereafter RMC), to identify areas, globally and in Australia, with a climate similar to location records of *T. mitsukurii* in its native range in Asia (China, Japan, South Korea, Taiwan, Thailand and Vietnam). The underlying assumption in this analysis is that the species can establish in climatically similar locations in the novel area [31,40,41,42,43,44]. However, there are known deficiencies in climate matching analyses [31,44,45]. It has been noted that a “milder” climate, with a smaller range of extremes, may show up as having a poor match to the climate from which the species originated, and that the insensitivity of the climate match index to the direction of difference for each climate parameter limits the usefulness of a climate matching analysis [45]. Erroneous location records can potentially have an impact on the climate matching analysis [44], as such an analysis does not consider the biology of the species.

Because our analysis highlighted the known deficiencies [31,44,45] of using climate matching to assess the potential distribution of a species, we then used the better-known and more robust CLIMEX Compare Locations model [43] to assess the potential distribution of *T. mitsukurii*, both in Australia and globally. As has been pointed out, the CLIMEX Compare Locations model is extremely robust and powerful because a variety of input data can be used for cross-validation [31,43,46].

The aims of this paper are to (a) use CLIMEX to assess the potential distribution of *T. mitsukurii* globally; (b) use CLIMEX to assess the potential range overlap of *T. mitsukurii* and *T. japonicus* with *H. halys* in Australia, in order to understand the climatic potential for these parasitoids to help mitigate the impact of *H. halys* in Australia; and (c) use the same analyses to consider the situation in Europe, where work is ongoing to assess the potential of both *T. mitsukurii* and *T. japonicus* as biological control agents for *H. halys* [22,47,48,49,50].

## 2. Materials and Methods

### 2.1. Location Records: Trissolcus mitsukurii

Location records for *T. mitsukurii* were obtained from Hymenoptera Online (HOL) [51] from a literature search [17,27] and from an examination of preserved specimens in China [52,53]. These various sources provided a total of 61 point location records and six polygon records for *T. mitsukurii* globally. All records in the final dataset were geocoded, with latitude and longitude coordinates. Duplicate records (multiple specimens recorded at the same location) were removed.

A record for Queensland (Charters Towers) [54] that was reclassified as *T. mitsukurii* [26] was found subsequent to the analyses, as was a record for *T. mitsukurii* in Iran [55]: these records were not included in any analyses, but are shown on the maps. The record in HOL for Hawaii (introduction in November 1966) was not included, as *T. mitsukurii* never established there [56].

### 2.2. Location Records: Trissolcus japonicus

Geocoded location records for *T. japonicus* were derived from HOL, from a recent examination of specimens in China [52,53], from the literature [13,16,17,18,48] and from colleagues [57,58]. A total of 99 point location records were obtained.

### 2.3. Location Records: Halyomorpha halys

Location records for *H. halys* were initially obtained from GBIF [59]. Records with no coordinates associated with them and duplicate records (identical latitudes and longitudes, representing different specimens from a single location, or multiple entries for the same record with the same event date and time) were removed. A number of records were removed because *H. halys* does not occur as established populations at these locations. This included records from New Zealand, Sweden, Iceland, Malta, Denmark and England, which reflect interceptions only: *H. halys* is not known to occur in these locations [60,61,62,63]. Records for Alaska were also removed, as these are likely interceptions since this region is considered too cold for the establishment of permanent populations [7]. A record for Manitoba in Canada was removed, since there are no established populations of *H. halys* there [64], as were three records from southern Florida, which were deemed to be interceptions [52].

Numerous records were acquired from colleagues (G Anfora, Fondazione Edmund Mach, Italy; A Caponero, ALSIA—Integrated Pest Management Service, Italy; S Castro, University of Coimbra, Portugal; TD Gariepy, Agriculture and Agri-Food, Canada; H Gaspar, University of Coimbra, Portugal; T Haye, CABI, Switzerland; J Loureiro, University of Coimbra, Portugal; D Li, Ministry for Primary Industries, New Zealand; L Maistrello, University of Modena and Reggio Emilia, Italy; N Meskhi, National Food Agency, Georgia; C Phillips, AgResearch, New Zealand). Records were also obtained from the literature [16,17,18,27,48,65,66,67,68] and from HOL (TY). For *H. halys*, a total of 14,529 point location records were obtained.

All datasets and associated shapefiles for these three species are now available on the International Pest Risk Research Group website (https://pestrisk.org/geographical-distribution-datasets-for-bmsb-and-two-of-its-parasitoids/).

### 2.4. Meteorological Data

We ran all CLIMEX modules with the CM10_1975H V1.1 CliMond climatic dataset [69]. This is a global 10′ gridded dataset, which comprises 30-year averages (1960–1990) of monthly values for daily minimum and maximum temperature (°C), relative humidity (%) at 09:00 and 15:00 and monthly rainfall total (mm).

### 2.5. CLIMEX Regional Match Climates: Trissolcus mitsukurii

The variables used in the Regional Match Climates (RMC) runs were minimum and maximum temperature and soil moisture. Other authors have used minimum and maximum temperature and rainfall total [44,70], and also included the rainfall pattern [42,71]. We opted to use soil moisture because *T. mitsukurii* is a parasitoid of several stink bug species, which are themselves pests of various agricultural and horticultural crops (rice, beans and orchard fruits) [72], and therefore, likely to be present in crops that are irrigated. Because this analysis indicated a low match (0.55) between the climates in the native distribution with known location records in Australia, we performed a reverse RMC analysis, whereby we compared the climates of the exotic locations (17 in Australia and 12 in Italy) to the rest of the world. Finally, we ran the RMC using the four standard factors (minimum and maximum temperatures, rainfall amount and pattern) [42,71].

As the RMC analyses were of limited value, full details of the methodology and results and a brief discussion of the results can be found in Appendix A.

### 2.6. CLIMEX Compare Locations

Because there are existing CLIMEX Compare Locations models available for *H. halys* [7] and *T. japonicus* [39], we constructed a CLIMEX Compare Locations model for *T. mitsukurii*. Only the Asian location records were used to fit the model, with the remaining exotic location records reserved for validation. As we specifically used the *T. mitsukurii* model with irrigation, we also ran the other models with the same irrigation scenario for comparison, although the original authors of those models did not include irrigation scenarios in their publications. Parameter values for all three models are provided in Table 1 for comparison, and details on fitting all parameter values for *T. mitsukurii* are provided below.

#### 2.6.1. Moisture Index

There was limited information with which to set these parameters for *T. mitsukurii*, save looking at the native distribution and knowing that *T. mitsukurii* is an egg parasitoid of stink bug species that are pests of various agricultural and horticultural crops [72]. Assuming that plant hosts are grown under favourable conditions, the lower soil moisture threshold (SM0) was set at 0.1 to reflect the permanent wilting point of plants [73]. The lower optimum soil moisture (SM1) was set at 0.5 because all hosts of *T. mitsukurii* occur on plants requiring a reasonable amount of water (e.g., rice). The upper optimum (SM2) was set at 1.6. *Trissolcus mitsukurii* parasitises eggs of *N. viridula*, the green vegetable bug, which in turn is a major pest and cause of pecky rice in Japan [72], and rice is cultivated in wet conditions. In Japan, the maximum soil moisture exceeds 1.6 in half of the grid cells, incorporating the known location records. The native range does not have to be 100% optimal; hence, setting SM2 at 1.6 does not preclude occurrence but reduces the growth potential when soil moisture exceeds this level. The upper threshold (SM3) was set at 1.8, to accord with the soil moisture profiles for the known wettest location records.

#### 2.6.2. Temperature Index

There were two sources of information on the development of *T. mitsukurii* [6,74], from experiments where *T. mitsukurii* was reared in egg masses of *H. halys* kept at different constant temperatures. In one study [6], the authors calculated a developmental threshold temperature of 11.7 °C for males and 11.8 °C for females; however, zero males and only 8% of females emerged at 15 °C. Furthermore, the zero emergence of males at 15 °C was not included in their calculation of the developmental threshold temperature for males. Using these published data, we calculated (using linear regression) the same value of 11.7 °C for males by excluding the zero value at 15 °C, but a value of 13.5 °C by including the zero development at 15 °C. For females, we calculated a developmental threshold of 11.6 °C, rather than 11.8 °C. With data for both sexes combined and the zero development of males at 15 °C excluded, we calculated the threshold temperature to be 11.8 °C, and with the zero data point included, we calculated the threshold to be 12.8 °C. In the second study [74], where *T. mitsukurii* was reared in temperatures from 20 °C to 28 °C, the authors calculated the developmental threshold to be 11.51 °C for females, 12.15 °C for males or 12.29 °C for males and females combined. As these calculations all suggest a developmental threshold between 11.5° C and 13.5 °C, DV0 was set to 12 °C.

The data from these experiments showed that the development rate increased linearly with increasing temperatures, development was fastest at 27.5 °C and the highest proportion of adults (93.4%) emerged at this temperature [6]. Therefore, 27.5 °C is not the maximum temperature at which development occurs, and this is supported by subsequent experiments on *T. mitsukurii* conducted at 25 ± 2 °C [72]. The optimal temperature was set to range between 25 °C (DV1) and 30 °C (DV2), effectively bracketing 27.5 °C.

The maximum temperature for development (DV3) was set at 32 °C. There is no information beyond 27.5 °C [6]; however, by definition, DV3 must be greater than DV2 (30 °C), and the maximum temperatures at the known locations for *T. mitsukurii* in Japan range from 26 °C–31.4 °C. The maximum temperature at the location record in Thailand is 37 °C, so having DV3 set at 32 °C will merely curtail the population growth of *T. mitsukurii* in the hottest part of the year in Thailand.

#### 2.6.3. Cold Stress

*Trissolcus mitsukurii* can clearly survive sub-zero temperatures, but minimum temperatures at known locations in Japan do not fall to −10 °C in winter. The temperature threshold Cold Stress (CS) parameter values (Table 1) restrict the polar distribution limits of *T. mitsukurii* in Asia.

In order to be thorough in our modelling process, the CLIMEX degree-day mechanism of CS was also examined. All combinations of parameters for this mechanism enabled persistence at unrealistically far northern regions. Because this mechanism did not limit the poleward distribution of *T. mitsukurii* in Asia, we did not include it in the final model.

#### 2.6.4. Heat Stress

With the temperature threshold mechanism of Heat Stress (HS), stress cannot accumulate at temperatures favourable to growth; hence, the temperature threshold (TTHS) at which stress begins to accumulate must be at or above the maximum temperature for development (DV3), (e.g, see [43,75,76]). Some HS may occur in the native range (i.e., at the Thai location where the maximum temperatures reach 37 °C (see above)), although clearly, it cannot be so severe as to preclude persistence. With TTHS at 32 °C (DV3), a very low stress accumulation rate (THHS = 0.001 week^−1^) provided excessive stress at the Thai location, precluding persistence here. Increasing TTHS to 33 °C and to 34 °C whilst maintaining the stress accumulation rate very low (at 0.001 week^−1^) reduced the HS and correspondingly increased the EI at this location. However, we had no basis for selecting one temperature threshold over another, and an accumulation rate of 0.001 week^−1^ is likely too low: if high temperatures *per se* are detrimental, a negative impact should occur relatively quickly, accompanied by a sharp decrease in development or survival with increasing temperatures.

We therefore tested the degree-day mechanism of HS, which enables HS to accumulate when the threshold number of degree-days above the developmental threshold (DV3 = 32 °C) is exceeded. This mechanism imposed some HS at the Thai location, whilst maintaining plausible suitability (EI = 12). Because this mechanism maintained a consistency in the temperature at which development ceases and stress impacts on survival, we opted to utilise this mechanism for the accumulation of HS.

#### 2.6.5. Dry Stress

The Dry Stress (DS) threshold was set at the lower soil moisture threshold for growth (SM0), and the rate of stress accumulation set reasonably high (0.01). The parameter values for DS (Table 1) provided no DS in Asia.

#### 2.6.6. Wet Stress

The Wet Stress (WS) parameters (Table 1) provided low levels of WS at one location in Japan (WS = 2) and at both locations in Taiwan (ranging from 5–18). Reducing WS by increasing the threshold soil moisture (SMWS) to 2 removed all WS at known locations. Whilst this marginally increased the EI of the Taiwanese locations (from 19 to 23 and from 14 to 15), it had no impact on the EI of the Japanese location. Without additional information, there was no compelling reason to reduce WS.

#### 2.6.7. Number of Generations (PDD)

The annual number of degree-days (°C days) above the developmental threshold of DV0 (PDD) represents the thermal accumulation needed to complete a full generation and for successful reproduction to occur. When the value of PDD exceeds the threshold, the excess contributes to the development of subsequent generations. One study [74] calculated that females needed 166 °C days above 11.5 °C and males required 146 °C days above 12.15 °C to complete development from oviposition to adult emergence. Another study [6] calculated that females required 206 °C days above 11.8 °C and males required 191 °C days above 11.7 °C to complete development from oviposition to adult emergence. Using meteorological data collected in Kochi city from May to October 1986 to 1996, this second study [6] estimated that males could accumulate 2900 and females 2882 °C days above their respective developmental thresholds. Hence, males could theoretically complete 15 generations, and females 14 generations, from 1 May to 31 October. Using the CliMond data [69], we found that the warmest of the three climate grid cells surrounding Kochi city accumulated a total of 2231 °C days above 12 °C, and 2306 °C days above 11.7 °C, and with PDD set at 190 °C days, this only permitted 11–12 generations per year from the end of February until mid-December (not just 1 May to 31 October). Regardless of whether DV0 was set at 12 °C or 11.7 °C, PDD needed to be reduced to 155 to allow 14 generations to be completed by the end of December. We set PDD at 185 °C days: this is slightly lower than the calculated value of 190 (for males) [6] to compensate for the slightly higher developmental threshold (DV0 set at 12 °C rather than 11.7 °C), but it still allows 12 generations to be completed in Kochi City.

#### 2.6.8. Model Runs

**Figure 1:**
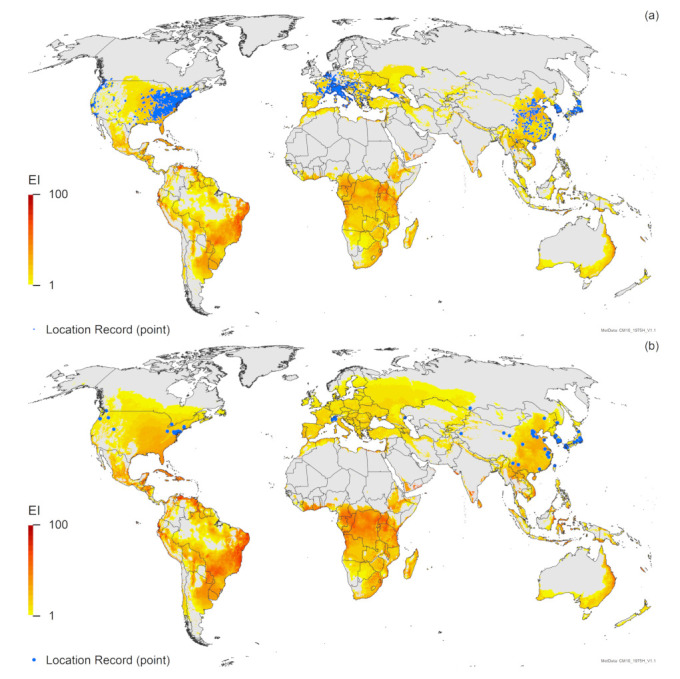

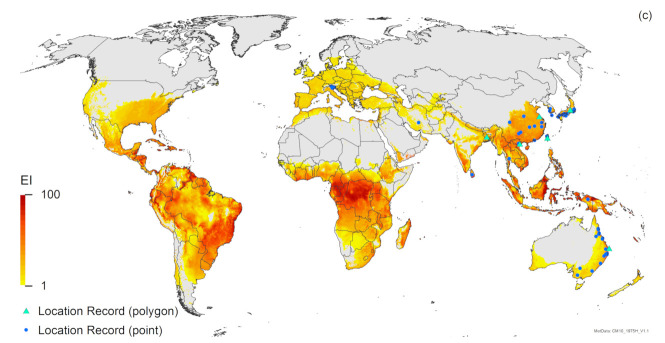
Modelled global climate suitability (CLIMEX Ecoclimatic Index) for (**a**) *Halyomorpha halys* [7], (**b**) *Trissolcus japonicus* [39] and (**c**) *Trissolcus mitsukurii.* Suitability for all three species is mapped as a composite of natural rainfall and irrigation based on identified irrigation areas [77]. Circles are point location records; triangles are polygon records.

We ran the model for the world using both natural rainfall and an irrigation scenario of 2.5 mm day^−1^ as top-up. We mapped our results using composite climate suitability maps, using identified irrigation areas [77]. For each 10′ cell, if the irrigated area was greater than 2.5 ha, the greater value of the natural rainfall and irrigation scenario results was used. Otherwise, the natural rainfall scenario result was used, e.g., Figure 1 in (e.g., Figure 1 in [78]).

## 3. Results

### 3.1. Regional Match Climates

The results of the RMC with three factors, comparing the native locations to the rest of the world are shown in Appendix A, Figure A3a,b. It is interesting that the Composite Match Index (CMI) for known locations of *T. mitsukurii* in Australia is relatively low: a value of only 0.55 still excludes a location record in Queensland (Appendix A, Figure A3b and Figure A4a).

A CMI of 0.7 from the standard RMC (with four factors) excludes most of Europe and still excludes two locations in Australia (Appendix A, Figure A3c and Figure A4b). A CMI of 0.65 in this analysis includes much broader regions of Australasia, Europe, Africa and South America (Appendix A, Figure A3d), but only excludes one Australian location record (Appendix A, Figure A4c). However, it includes the much broader regions of Australasia, Europe, Africa and South America (Appendix A, Figure A3d).

**Figure A2:**
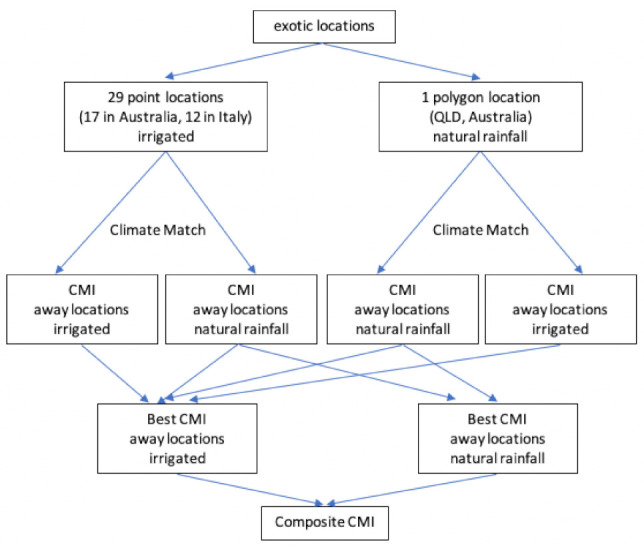
Schematic diagram of methodology for “Reverse” Climate Match (Regional) modelling.

The results of the reverse RMC, comparing the exotic locations (Australia and Italy) to the rest of the world, are shown in Appendix A, Figure A5. In accordance with the previous RMC analyses, a CMI of 0.7 excludes one quarter of the native locations (8 out of 32), although there are more regions globally with a climate similar to the exotic (Australian and Italian) location records than to the native range (Appendix A, Figure A5a vs. Figure A2a). The CMI cut-off needs to be decreased to 0.60 to include all Asian location records, resulting in a much larger area globally with a similar climate (Appendix A, Figure A5b).

**Figure A3:**
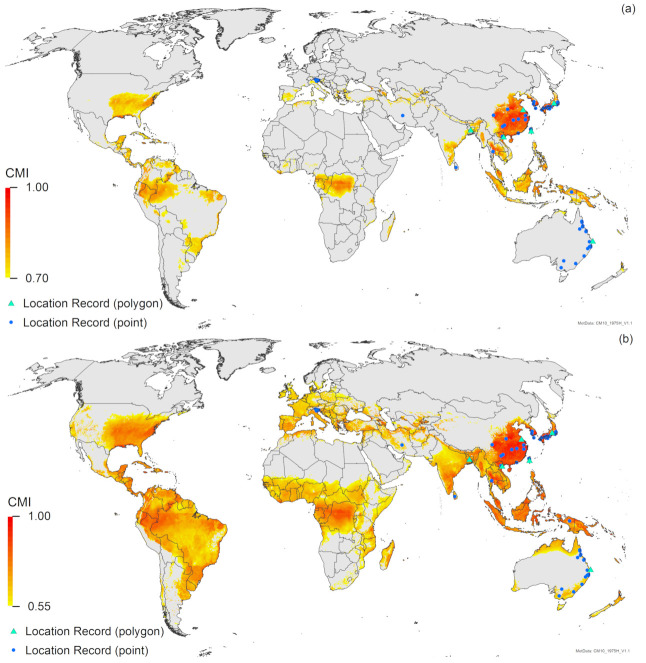

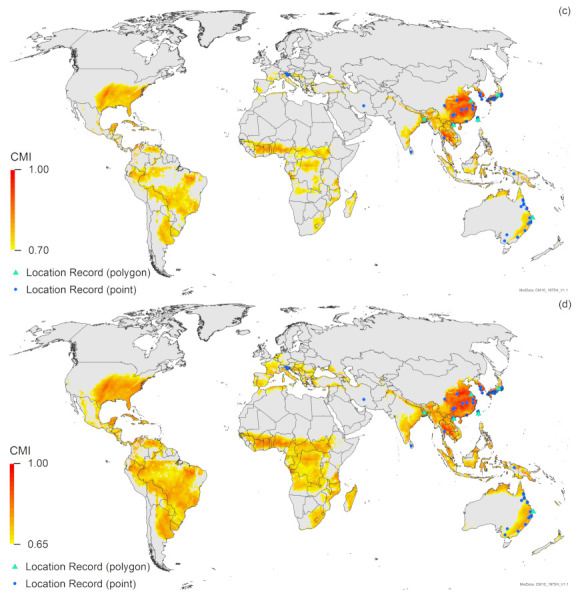
Composite map of the maximum Climate Match Index (CMI) between native locations and the rest of the world: (**a**) Regional Match Climate (RMC) using minimum and maximum temperature and soil moisture, CMI > 0.70; (**b**) RMC using minimum and maximum temperature and soil moisture, CMI > 0.55; (**c**) RMC using minimum and maximum temperature, rainfall amount and pattern, CMI > 0.70; (**d**) RMC using minimum and maximum temperature, rainfall amount and pattern, CMI > 0.65. Location records are for *Trissolcus mitsukurii*: circles are point location records; triangles are polygon records.

The RMC analyses where both Asian and Australian location records are considered to be the native range of *T. mitsukurii* are presented in Appendix A, Figure A6. The analyses using soil moisture (three factors) and rainfall (four factors) differ, although not surprisingly, a CMI of 0.7 includes all location records in both Asia and Australia in both analyses. The RMC using soil moisture (Figure A6a) projects less of the world as having climates that match the climates of the Australian and Asian locations than does the RMC using the rainfall factors (Figure A6b). These analyses show different results compared to the previous ones (i.e., Figure A3a and Figure A5a versus Figure A6a and Figure A3c versus Figure A6b).

### 3.2. Compare Locations

Having fitted the parameter values to the Asian location records, we ran the model for the world. All exotic location records (Australia and Italy, our validation records) fell within regions projected to be climatically suitable, as did the two records found just prior to submission of this paper.

Results from the Compare Locations runs on all species (*H. halys*, *T. japonicus* and *T. mitsukurii*) show a high degree of spatial concordance amongst the modelled potential distributions of these three species (Figure 1). This is not surprising, as the three species co-occur in their native range. *Halyomorpha halys* is established on four continents, but its projected potential distribution also includes large portions of Africa and Australasia, where it does not currently occur. While *H. halys* is already widespread in a number of areas in its adventive distribution in Europe, North and South America, the current distribution encompasses only a small portion of the projected potential distribution, especially in South America (Figure 1a). The two *Trissolcus* species have projected distributions for most of Europe, with *T. japonicus* projected to have a distribution significantly more extensive than that of *T. mitsukurii*, extending further north into Scandinavia and Russia (Figure 1).

As we used a top-up irrigation scenario for all the model runs, it is worth noting that there are minor differences in the projections shown here for both *H. halys* and *T. japonicus*, compared to the original models for these species [7,39]. Most noteworthy is that the irrigation scenario makes parts of mid-western North America and northern and western regions of China suitable for both *H. halys* and *T. japonicus*. New location records for both of these species (i.e., not used in the previous analyses [7,39]) fall within these regions, suggesting that irrigation may have enabled the spread of both species.

The Compare Locations analysis suggests that many of the areas projected to be suitable for *T. mitsukurii* (Figure 1c) are in regions that have similar climates to those where *T. mitsukurii* has been found (Match Climates analysis, Appendix A, Figure A3b,d and Figure A5b).

**Figure 2:**
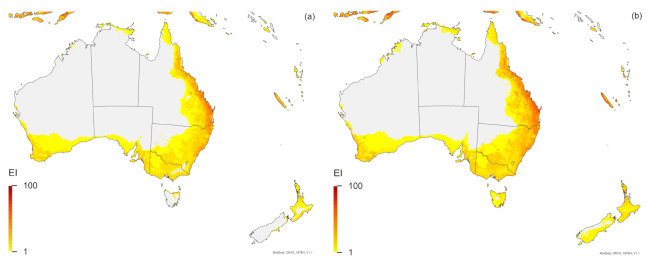

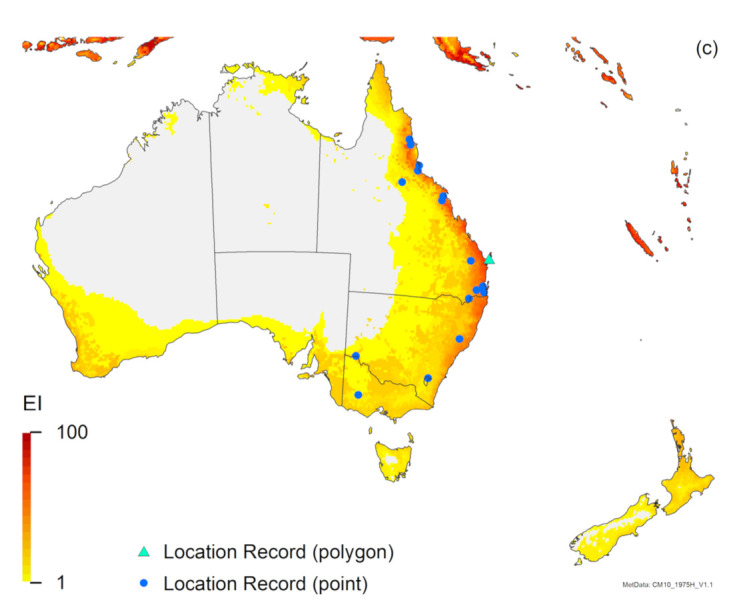
Modelled climate suitability (CLIMEX Ecoclimatic Index) for (**a**) *Halyomorpha halys*, (**b**) *Trissolcus japonicus* and (**c**) *Trissolcus mitsukurii* in Australia and New Zealand, modelled using CLIMEX Compare Locations. Suitability for all three species is mapped as a composite of natural rainfall and irrigation based on identified irrigation areas [77]. Circles are point location records; triangles are polygon records.

**Figure 3:**
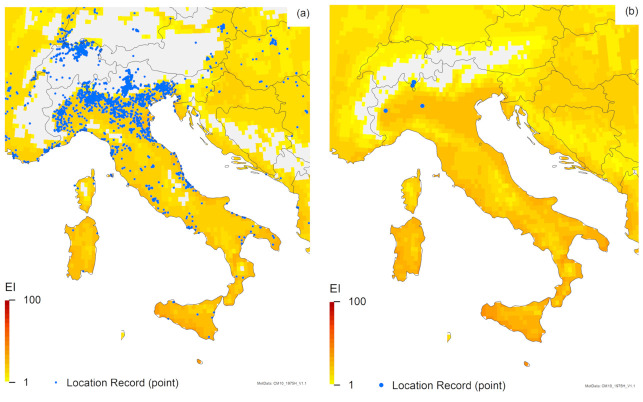

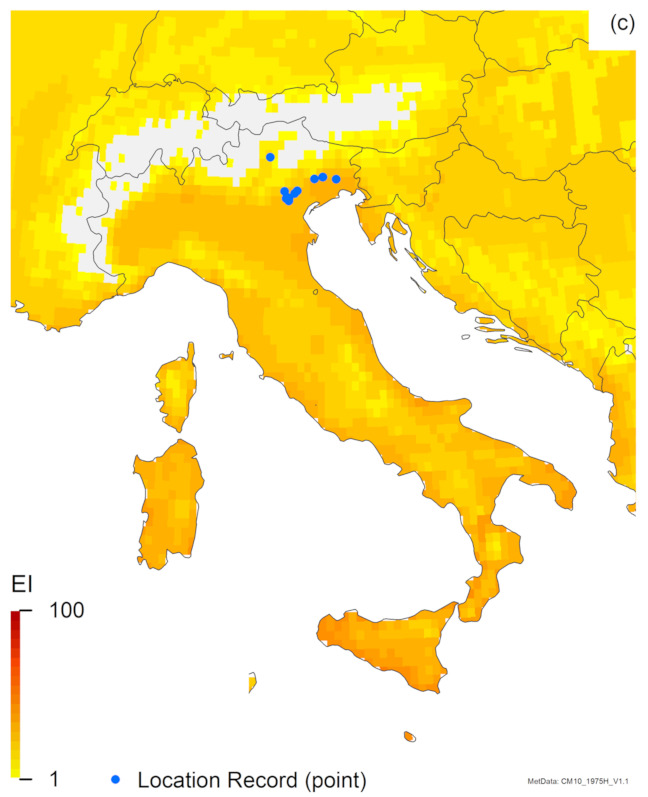
Modelled climate suitability (CLIMEX Ecoclimatic Index) for (**a**) *Halyomorpha halys*, (**b**) *Trissolcus japonicus* and (**c**) *Trissolcus mitsukurii* in Italy. Suitability for all three species is mapped as a composite of natural rainfall and irrigation based on identified irrigation areas [77].

For Australia and New Zealand, the two *Trissolcus* species have similar modelled potential distributions, but the potential distribution of *T. mitsukurii* in Australia is expected to range further inland, and in New Zealand, the North Island is projected to be marginally more suitable for *T. mitsukurii* than for *T. japonicus* (Figure 2). All known locations in the non-native (exotic) range fall within areas projected by the model to be suitable for *T. mitsukurii* (Figure 1c), with EI values ranging from 1 to 59 in Australia (Figure 1c and Figure 2c) and ranging from 19 to 30 in Italy (Figure 1c and Figure 3c).

The projected range overlap of all three species is closely aligned (Figure 1, Figure 2, Figure 3). In South America, Australia, Italy and perhaps all of Europe, *H. halys* is projected to have the most limited potential distribution from all three species modelled (Figure 1, Figure 2, Figure 3), whilst in North America, *T. mitsukurii* is projected to have the most limited potential distribution (Figure 1).

## 4. Discussion

Both the Match Climates and Compare Locations analyses suggest that *T. mitsukurii* could spread to many parts of the world where it does not currently exist. Whilst there are differences in the regions with climates similar to the native (Figure A3b,d) and exotic (Figure A5b) ranges of *T. mitsukurii*, there is nonetheless a high degree of overlap, suggesting that the climates in these areas are suitable for this species. The Compare Locations analysis (Figure 1c) suggests a compromise between the two Match Climates analyses (Figure A3b and Figure A5b), yet still suggests that *T. mitsukurii* has the potential to vastly expand its current distribution. All analyses of the potential distribution of *T. mitsukurii* suggest that this species will be climatically suited to most areas where *H. halys* currently occurs or may spread (Figure 1a).

The RMC analysis merely indicates the similarity of climates in different areas; hence, it is not surprising that most of Europe shows up as having a climate similar to that of the exotic locations (Italy and Australia, Figure A5). This “reverse” RMC suggests that it is likely that *T. mitsukurii* will continue to spread throughout Europe, to other areas with a climate similar to that of northern Italy, where it already occurs [17,27,48,79]. As the climate in the exotic range of *T. mitsukurii* is clearly suitable for the species, it stands to reason that the higher degree of similarity in climates worldwide to the exotic locations (Figure A5) rather than to the native locations (Figure A3) is a more appropriate indication of the potential area globally suitable for this species. This analysis suggests that *T. mitsukurii* can tolerate a broader climate space than indicated by the location records in its native range. To our knowledge, this is the first time a “reverse” Climate Match has been done for a species, to use the climate in the invaded range to assess the potential for the species to establish elsewhere. However, due to limitations inherent in climate matching analyses (see Appendix A), we focus instead on the Compare Locations analysis.

**Figure A4:**
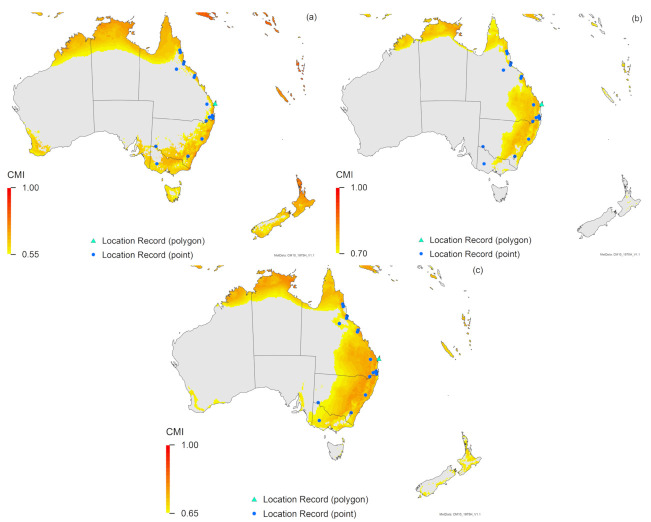
Composite map for Australia and New Zealand of the maximum CMI between native locations and the rest of the world: (**a**) Regional Match Climate (RMC) using minimum and maximum temperature and soil moisture, CMI > 0.55; (**b**) RMC using minimum and maximum temperature, rainfall amount and pattern, CMI > 0.70; (**c**) RMC using minimum and maximum temperature, rainfall amount and pattern, CMI > 0.65. Location records are for *Trissolcus mitsukurii*: circles are point location records; triangles are polygon records.

**Figure A5:**
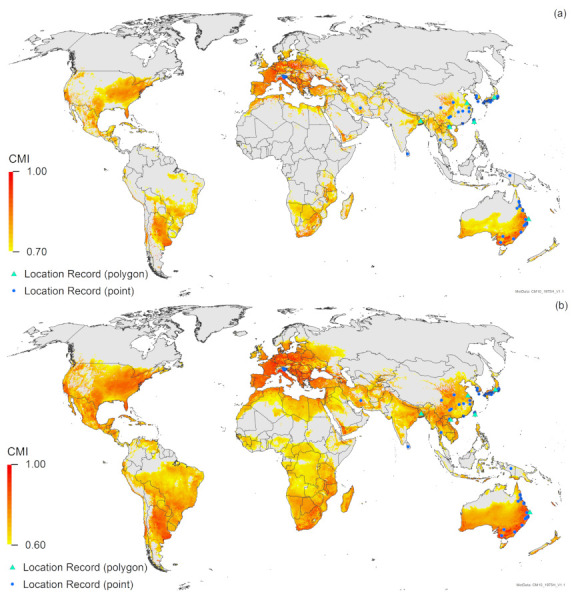

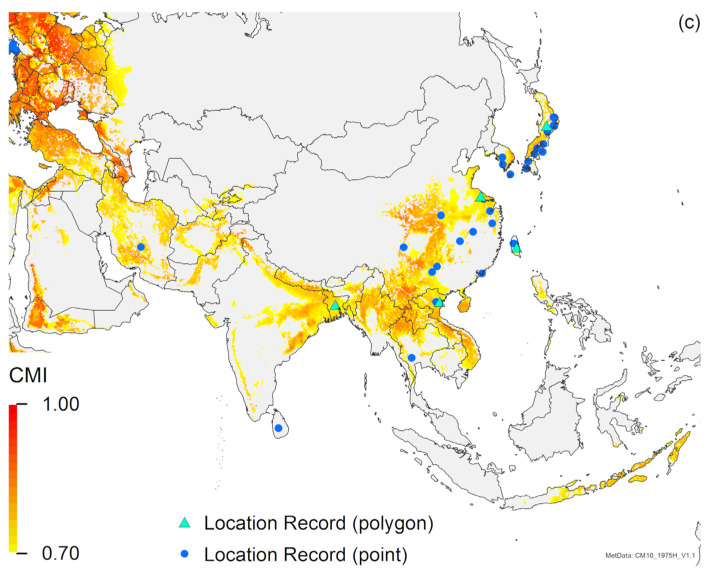
Composite map of the Regional Match Climate (RMC) of exotic irrigated locations from which *Trissolcus mitsukurii* has been recorded, compared to the world, using minimum and maximum temperature and soil moisture: (**a**) CMI > 0.70; (**b**) CMI > 0.60; (**c**) CMI > 0.70 for Asia only. Location records are for *Trissolcus mitsukurii*: circles are point location records; triangles are polygon records.

**Figure A6:**
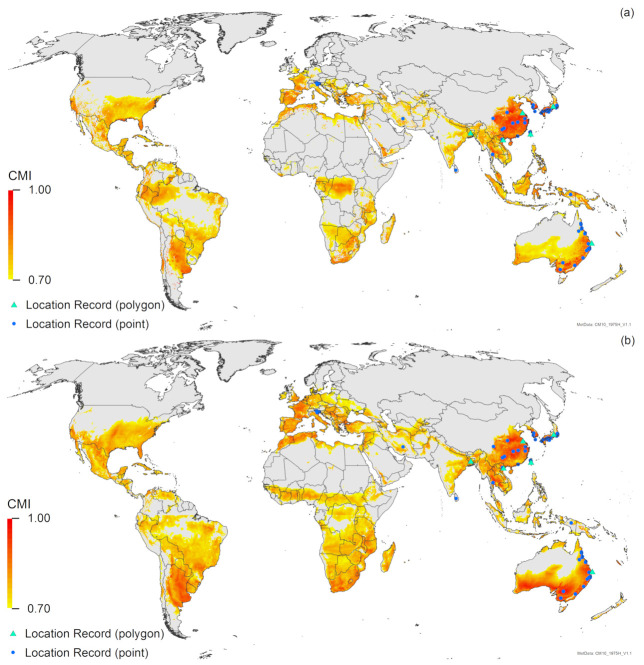
Composite map of the maximum Climate Match Index (CMI) between Asian and Australian locations and the rest of the world: (**a**) Regional Match Climate (RMC) using minimum and maximum temperature and soil moisture, CMI > 0.70; (**b**) RMC using minimum and maximum temperature, rainfall amount and pattern, CMI > 0.70. Location records are for *Trissolcus mitsukurii*: circles are point location records; triangles are polygon records.

In the RMC analyses, we initially consider exotic (Italy and Australia) location records separately from native (Asia) records. We provide our rationale for considering *T. mitsukurii* to be a naturalised exotic in Australia (there is no contention that it is an exotic species in Italy), recognising that our presumption may not accord with the views of all researchers. However, we summarise the current state of knowledge (which is to say, there is no real information as to the status of *T. mitsukurii* in Australia), we state the rationale behind our decision to treat Australian location records as naturalised exotic (acknowledging that this is not a statement of fact, but a position we have taken in regards to our analysis of location records) and we clearly state that our presumption of it being a naturalised exotic in Australia can and should be tested via population genetics studies. We tested whether our presumption that *T. mitsukurii* is a naturalised exotic in Australia affects our models and their projections. If *T. mitsukurii* is eventually classified as native to Australia, then the RMC analyses in Figure A3, Figure A4 and Figure A5 would be incorrect, but the results shown in Figure A6 would hold. The differences reflect the fact that Italy does not have the warm wet conditions found in north-eastern Australia or the south-eastern part of the native range in China. Nonetheless, all RMC analyses indicate that *T. mitsukurii* has the potential to spread well beyond its current distribution, and as we discuss in the Appendix, climate-matching analysis is considered to be a more simplistic approach [31,43] and has well-known limitations [42,45,80]. For the Compare Locations analysis, we used Asian locations to parameterise the model, and exotic (Italian and Australian) locations to validate the model. As all the validation records fall within areas projected to be suitable, there is no consequence of our assumption that *T. mitsukurii* is a naturalised exotic in Australia. The model will only need to be revisited and adjusted if *T. mitsukurii* is ever found in regions falling outside the ranges projected to be suitable.

**Figure 4:**
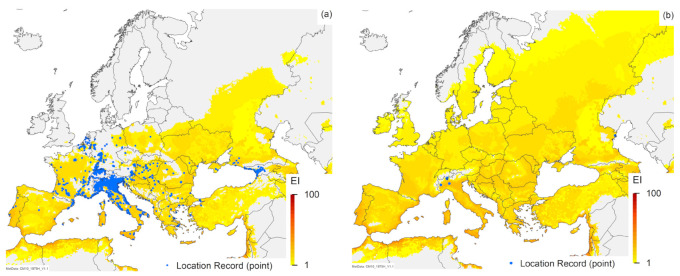

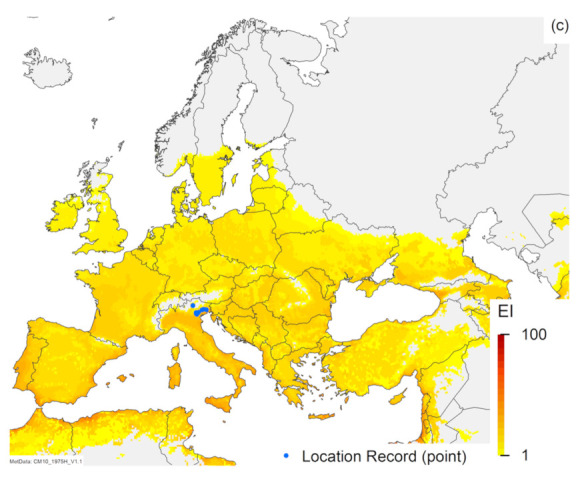
Modelled climate suitability (CLIMEX Ecoclimatic Index) for (**a**) *Halyomorpha halys*, (**b**) *Trissolcus japonicus* and (**c**) *Trissolcus mitsukurii* in Europe modelled using CLIMEX Compare Locations. Suitability for all three species is mapped as a composite of natural rainfall and irrigation based on identified irrigation areas [77].

The Compare Locations CLIMEX model fitted to the Asian locations accords with all known exotic locations, and suggests that *T. mitsukurii* could expand its distribution considerably, particularly in South America, Africa and Australasia. Serendipitously, the two data points (Iran and Australia) found subsequent to the analyses both fall within areas projected to be climatically suitable (Figure 1c and Figure 2c), as do all new location records for Italy [79], providing some measure of confidence in the model. The modelled potential distribution of *T. mitsukurii* is closely aligned to those projected for *H. halys* and *T. japonicus* (Figure 1, Figure 2, Figure 3, Figure 4). It is encouraging that the projections all suggest that singly or together, both *Trissolcus* species will be able to occupy the same climate areas as *H. halys*, and thus, in conjunction have the potential to control this pest throughout its range globally. Work is underway in Australia to better identify the current range of *T. mitsukurii*, which will help increase our understanding of the climatic tolerances of this species.

It is difficult to find much difference between the current projections for *T. japonicus* with irrigation (Figure 1b) and without [39]. A few more regions in northern and eastern China are made suitable with irrigation, and three location records not part of the original analysis [39] are located in these regions. An increased area of western North America is projected to be suitable with irrigation, but the new location records obtained for this analysis probably fall within the areas projected as suitable without irrigation. New location records for *T. japonicus* in Europe [79] further validate that model [39], as they all fall within areas projected to be suitable.

Similarly, there are minimal differences between the projections for *H. halys* in this analysis, which includes irrigation (Figure 1a), and the non-irrigated simulations published previously [7]. However, the composite map (Figure 1a) shows a few more regions of western China as being suitable for *H. halys*, and it is interesting to note that two additional location records in this paper fall in these areas, designated as suitable due to the addition of irrigation in these model runs. New location records for Thailand, Malaysia, Java and North America (Figure 1a) all fall in areas projected to be suitable, as do most new records in Europe (Figure 3a and Figure 4a), lending further support for this model [7]. However, there are new location records for *H. halys* in Europe that do not fall within areas projected to be climatically suitable (Figure 3a and Figure 4a). As demonstrated in a recent analysis for Switzerland [38] using a finer climate grid (0.02° versus the 0.1667° of the CliMond dataset used here), this is likely due to the scale of the climate grid rather than an issue with the CLIMEX model. The location records in areas projected to be unsuitable in Italy, Switzerland, Austria and Germany (Figure 3a and Figure 4a) all fall in mountainous areas with high topographic relief, where the modelled climate is not represented at sufficient granularity to match the distribution records appropriately [81]. Ten of the new records in the Netherlands fall in areas projected to be climatically unsuitable (Figure 4a) due to an insufficient heat sum (growing degree-days) in which to complete a generation [7]. The model [7] is likely to be correct, as the degree-day requirement was based on numerous studies [82,83,84]. Examination of these records shows that they are all in built-up areas, and were generally found on buildings, and all but one of the records are of adults (one is of a nymph). Thus, either these records indicate transient individuals, as *H. halys* is capable of long-distance flight [3,85,86], or heating of buildings is providing suitable microclimates that enable *H. halys* to persist in otherwise unfavourable conditions (e.g., the nymph record, collected in the corner of a building). The true status of *H. halys* in the Netherlands needs to be confirmed, as it is not yet clear whether current records indicate transient populations or breeding populations.

This highlights that any bioclimatic modelling approach is limited because only climatic variables are included. Non-climatic effects (e.g., host availability, behaviour, competition, predation, habitat preference and landscape features) can also influence the distribution of a species. This is clearly demonstrated by the anomalous *H. halys* location records in the Netherlands. However, because CLIMEX parameter values are set by the user, it is possible to then look at such records, understand why the location is being projected to be climatically unsuitable, and then postulate reasons (that may easily be explored and investigated) that nonetheless enable the species to establish. Where a suitable covariate is available, such as the GMIA to indicate the presence of irrigation, it is possible to incorporate such climate-modifying effects into the modelling workflow [76].

All of our analyses (Figure 1, Figure 2, Figure 3, Figure 4) indicate that there is a high degree of overlap in the projected global ranges of *H. halys*, *T. japonicus* and *T. mitsukurii*. Given that all three species coexist in Asia, this is not surprising. In North America, *T. japonicus* is likely to be able to occupy the same climate space as *H. halys* (Figure 1, Figure 3 and Figure 4), and *T. japonicus* has in fact spread in North America compared to the 2018 study [39]. Both *T. japonicus* and *T. mitsukurii* have a slightly broader projected distribution in Europe than *H. halys* (Figure 1, Figure 3 and Figure 4), and so far, both species have been found to be rapidly expanding their ranges alongside *H. halys* [79]. It therefore appears that the two *Trissolcus* species should, in combination, help mitigate the impacts of *H. halys* as it continues its inexorable invasion of new areas.

To further refine our model, future research on *T. mitsukurii* needs to focus most urgently on two areas: phenology and geographical range. It would be useful to obtain geocoded phenological data on *T. mitsukurii* in Taiwan, to validate the soil moisture parameters in the model. Currently, the upper threshold (SM3) is set at 1.8, in accordance with the soil moisture profiles for the known location records. Both location records in Taiwan fall within irrigated areas in the GMIA [77], and although there is no irrigation in the wet season, modelled growth of *T. mitsukurii* is prevented from mid-May until mid-August because high rainfall causes the soil moisture to exceed 1.8. Capturing and understanding the phenology of *T. mitsukurii* populations in such wet areas would assist in model validation, as well as help us understand the climatic tolerances of this species. Additional research is needed in other areas to identify when *T. mitsukurii* is active and growing. It has been estimated that 14–15 generations are possible at Kochi City from May to October, and it has been suggested that other hosts must be used since *H. halys* is univoltine or bivoltine in Japan [6]. Our CLIMEX model indicates that population growth of *T. mitsukurii* occurs from the end of February until the end of December (not just May to October), but that only 12 generations are possible. These discrepancies could be due to our use of long term average climate data, rather than daily meteorological data collected over a much shorter timeframe [6]. However, as this is the only study available with some information on the phenology of *T. mitsukurii*, additional phenological data from other locations would serve to cross-validate the model.

Because *T. mitsukurii* has now already been found well outside its native range in Asia, it may be more relevant to assess its range in these new regions. In particular, determining the true extent of the range of *T. mitsukurii* in Australia would provide valuable information in preparation for the likely establishment of *H. halys*. It is currently unlikely that Australia will be able to import *T. japonicus* to assist in the control of *H. halys*, and thus, whilst *T. japonicus* may ultimately invade Australia, *T. mitsukurii* may well be the initial frontline defence against *H. halys*. The widespread distribution of *N. viridula* (www.ala.org.au, accessed on 21 February 2021, data not shown) accords closely with the modelled potential distribution of *T. mitsukurii*, including all of the horticultural areas in southern Australia, the south-west of Western Australia, the arable lands in South Australia and the horticultural areas of Tasmania, from which we have no records of *T. mitsukurii*. Given that *T. mitsukurii* has invaded Italy, it seems unlikely that it could not have spread throughout Australia in the areas modelled as being climatically suitable and which are known to include at least one host. Parasitoids such as *T. mitsukurii* are very small and likely will not be recognised unless actively sought out; hence, we need to be careful of confusing an absence of evidence with evidence of absence when interpreting its present known distribution in Australia. It may well be that serendipitously, our first line of defence against *H. halys* is already mobilised and deployed.

## Figures and Tables

**Table 1:** CLIMEX parameter values for *Trissolcus mitsukurii*, *Trissolcus japonicus* [39] and *Halyomorpha halys* [7].

Parameter	Description	*T. mitsukurii*	*T. japonicus*	*H. halys*
**Moisture**				
SM0	lower soil moisture threshold	0.1	0.1	0.1
SM1	lower optimum soil moisture	0.5	0.4	0.5
SM2	upper optimum soil moisture	1.6	1.2	1
SM3	upper soil moisture threshold	1.8	1.6	1.5
**Temperature**				
DV0	lower temperature threshold	12 °C	12 °C	12 °C
DV1	lower optimum temperature	25 °C	27 °C	27 °C
DV2	upper optimum temperature	30 °C	30 °C	30 °C
DV3	upper temperature threshold	32 °C	34 °C	33 °C
**Diapause**				
DPD0	diapause induction daylength			12 h light
DPT0	diapause induction temperature			5 °C
DPT1	diapause termination temperature			5 °C
DPD	diapause development days			0
DPSW	diapause summer (1) or winter (0)			0
**Cold Stress**				
TTCS	cold stress temperature threshold	−7 °C	−18.3 °C	−18 °C
THCS	temperature threshold stress accumulation rate	−0.01 week^−1^	−0.0015 week^−1^	−0.01 week^−1^
DTCS	degree-day cold stress threshold			
DHCS	degree-day cold stress accumulation rate			
**Heat Stress**				
TTHS	heat stress temperature threshold		34 °C	33 °C
THHS	temperature threshold stress accumulation rate		0.055 week^−1^	0.01 week^−1^
DTHS	degree-day heat stress threshold	4		
DHHS	degree-day heat stress accumulation rate	0.0008 week^−1^		
**Dry Stress**				
SMDS	soil moisture dry stress threshold	0.1	0.1	0.1
HDS	stress accumulation rate	−0.01 week^−1^	−0.01 week^−1^	−0.01 week^−1^
**Wet Stress**				
SMWS	soil moisture wet stress threshold	1.8	1.6	1.5
HWS	stress accumulation rate	0.01 week^−1^	0.0065 week^−1^	0.002 week^−1^
**Hot Wet Stress**				
TTHW	hot wet stress threshold temperature		27 °C	28 °C
MTHW	hot wet stress threshold soil moisture		1.4	1.5
PHW	hot wet stress accumulation rate		0.0024 week^−1^	0.007 week^−1^
**Threshold Heat Sum**				
PDD	number of degree-days above DV0 needed to complete one generation	185 °C-days	175 °C-days	595 °C-days
**Irrigation Scenario**	2.5 mm week^−1^ as top up	all models run with this irrigation scenario in this paper		

Values without units are for a dimensionless soil moisture index, scaled from 0 (oven dry), with 1 indicating field capacity. A value of 0.1 is approximately the permanent wilting point.

**Figure A1:**
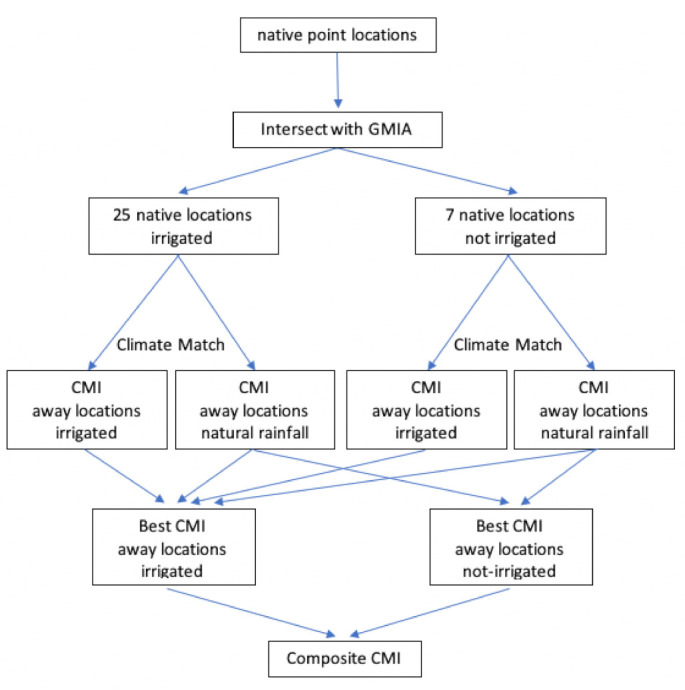
Schematic diagram of methodology for Climate Match (Regional) modelling.

## Data Availability

Location record spreadsheets and shapefiles are available on the IPPRG website.

## Appendix A. CLIMEX Match Climates (Regional) for *T. mitsukurii*

### Appendix A.1. Methods

The known location records for *T. mitsukurii* were separated into two shapefiles of native (Asia) and exotic (Italy and Australia) distributions. An initial assessment of the distribution records for *T. mitsukurii* revealed that some of the records were found in locations that were semi-arid, and were likely associated with plant hosts that support its pentatomid hosts. Because the plant hosts are likely supported in these areas by irrigation, it was necessary to consider the effects of irrigation explicitly in any climate matching exercise. Accordingly, the distribution records were intersected with the GMIA [77] to separate them into those that fell in irrigated and non-irrigated areas. This provided three shapefiles of point location records: native irrigated, native non-irrigated and exotic irrigated.

If a location record fell at the boundary of 2 (or 4) grid cells, all grid cells were used in the analysis. One location in Japan (Kochi, Muroto, Muroto-zaki (or Murotomisaki), lat 33.27910, long 134.17267, ID 18 in the non-irrigated shapefile) fell in the non-irrigated areas of the GMIA but outside of any grid cell in the CliMond CM10 dataset [69]; thus, it was not included in the Climate Match run, although it was included in the mapping.

The variables used in the Regional Match Climates (RMC) runs were minimum and maximum temperature and soil moisture. Other authors have used minimum and maximum temperature and rainfall total [44,70] and also included rainfall pattern [42,71]. We opted to use soil moisture because *T. mitsukurii* is a parasitoid of *H. halys*, a horticultural pest likely to be present in crops that are irrigated. Using the soil moisture parameter enabled us to use a plausible top-up irrigation scenario of 2.5 mm day-1 to both native and exotic locations. We weighted the three parameters equally, with a value of 1.

We added irrigation to native locations falling within the GMIA irrigated areas in order to compare them to the rest of the world. For native locations outside the GMIA irrigated areas, we did not apply irrigation when we compared these to the rest of the world. For both sets of locations, we compared the native locations to non-irrigated and irrigated “away” locations. To create our composite maps, we selected the highest Composite Match Index (CMI) possible from the four output files for each “away” grid cell that fell within the GMIA irrigated areas, based on the assumptions that (a) 100% of a cell may not necessarily be irrigated, and that (b) irrigation may not happen for the particular crop that *H. halys* attacks at that location, although it may happen for other crops. Hence, we wanted the maximum value possible for that cell. For “away” cells that fell outside the GMIA irrigated areas (i.e., agriculture occurs under natural rainfall), we selected the maximum value from runs where locations were not irrigated, regardless of the irrigation status of the native locations (see Figure A1). We did not include natural rainfall scenarios for home locations for the 25 native range records that fell in cells in the GMIA where irrigation was practiced. Our rationale here was that the plant hosts of *H. halys* and *N. viridula* in the native range would be irrigated if they were in a semi-arid environment.

Because the results of this analysis indicated a relatively low match (0.55) between the climates in the native distribution with known location records in Australia, we performed a “reverse” RMC analysis, whereby we compared the climates of the exotic locations (17 in Australia and 12 in Italy) to the rest of the world. Since all locations fell within the GMIA irrigated areas, we applied top-up irrigation to the exotic locations when comparing them to both irrigated and non-irrigated locations in the rest of the world (Figure A2).

A single location in Australia did not fall within the GMIA irrigated areas as it is on Fraser Island, a World Heritage National Park in Queensland. This particular sample was collected on 13 Oct 1978 with no sampling protocol specified; was identified by NF Johnson in 1990; was associated with *Biprorulus bibax* (spined citrus bug); was of undetermined sex; associated field notes specify “Ex-Casuarina; Camp A”; and the location is only identified to polygon level (i.e., to Fraser Island), i.e., it was not a geo-coded point location (HOL). *Biprorulus bibax* (Pentatomidae: Hemiptera) is native to Australia, feeding off indigenous citrus species, and is now a major pest of commercial citrus [87] and although it occurs in Queensland, New South Wales, Victoria and South Australia, it has not been recorded on Fraser Island (Atlas of Living Australia). Given the data associated with this record, it is most likely that this was a temporary incursion, perhaps brought in by tourists, rather than reflective of a local population. Although we do not know if there are any native hosts for *B. bibax* on Fraser Island, we nonetheless ran the RMC to compare this non-irrigated exotic location to the rest of the world, both irrigated and not irrigated (Figure A2).

Because of the low CMI (0.55) needed to include all known location records in either of the above RMC analyses, we ran the RMC as described above, but using the four standard parameters (minimum temperature, maximum temperature, rainfall total and rainfall pattern), all weighted equally (to 1). No irrigation scenarios were used in this run, since the irrigation scenario only affects the soil moisture factor, and does not affect either of the rainfall factors used in these analyses.

To test the effect of our assumption that *T. mitsukurii* is exotic to Australia, we performed RMC analyses by combining the Asian and Australian data, and treating only the Italian data [17,27] as exotic. We ran the RMC using minimum and maximum temperatures and soil moisture (equally weighted to 1) so we could examine any impacts of irrigation, and we then ran it using the standard four parameters of minimum and maximum temperature, rainfall amount and pattern (equally weighted to 1).

### Appendix A.2. Results

The results of the RMC, using minimum and maximum temperatures and soil moisture as equally weighted parameters, comparing the native locations to the rest of the world, are shown in Figure A3a,b. The higher the CMI, the more closely the climate of the away location matches that of the home (native) location, and hence, the potentially better suited that away location is for the species. It is interesting that the CMI for known locations of *T. mitsukurii* in Australia is relatively low: A value of only 0.55 still excludes two location records (one of which was found post-analysis) in Queensland (Figure A3b and Figure A4a), although a CMI of 0.55 includes the record for Iran found post-analysis (Figure A3b). Normally, a CMI > 0.7 would be considered the minimum for the successful establishment of a species [42,71]; however, this threshold CMI applies to when the four standard parameters are used in a Match Climates analysis. When fewer variables are used in an analysis, a higher threshold would be expected for a biologically meaningful comparison.

Again, a CMI of 0.7 from a standard RMC (with four parameters) excludes most of Europe and still excludes three locations in Australia, including the record found post-analysis (Figure A3c and Figure A4b). Whilst a CMI of 0.65 in this analysis still excludes one Australian location record (Figure A4c), it nonetheless includes much broader regions of Australasia, Europe, Africa and South America (Figure A3d).

The results of the RMC comparing the Australia and Italy to the rest of the world are shown in Figure A5. In accordance with the previous RMC analysis, a CMI of 0.7 indicates that there are more regions globally with a climate similar to the Australian and Italian location records than to the Asian range (Figure A5a vs. Figure A3a). However, a CMI of 0.7 excludes a quarter of the Asian location records (Figure A5c), and the CMI cut-off needs to be decreased to 0.60 to include all Asian location records, resulting in a much larger area globally with a similar climate (Figure A5b).

The RMC analyses where both Asian and Australian location records are considered to be native are presented in Figure A6. As before, the RMC analysis using soil moisture (and thereby allowing consideration of irrigation) and the analysis using the rainfall factors are somewhat different. The RMC using soil moisture (Figure A6a) projects less of the world as having have climates that match the climates of the Australian and Asian locations than does the RMC using the rainfall factors (Figure A6b). A CMI of 0.7 includes all location records in both Asia and Australia in both analyses.

### Appendix A.3. Discussion

The RMC with three factors (minimum and maximum temperatures and soil moisture) comparing the native locations with the rest of the world shows a relatively low match outside of Asia, even with a CMI threshold of 0.55 (Figure A3b), and excludes two location records for *T. mitsukurii* in Australia, even when these locations are irrigated (Figure A4a). The RMC comparing the climates of all exotic locations with the rest of the world, again using the same three factors, shows overall higher global CMIs as well as a larger area with a similar climate (Figure A5). As the RMC analysis merely indicates the similarity of climates in different areas, it is not surprising that most of Europe has a climate similar to that of the exotic locations (Figure A5). This “reverse” RMC suggests that it is likely that *T. mitsukurii* will continue to spread throughout Europe, to other areas with a climate similar to that of northern Italy, where it already occurs [17,27,48,79]. As the climate in the exotic range of *T. mitsukurii* is clearly suitable for the species, it stands to reason that the higher degree of similarity in climates worldwide to the exotic locations (Figure A5) rather than to the native locations (Figure A3) is a more appropriate indication of potential area globally suitable for this species. This analysis suggests that *T. mitsukurii* could in fact occur in a broader climate space than it experiences in its native range. To our knowledge, this is the first time a “reverse” Climate Match has been done for a species, to use the climate in the invaded range to assess the potential for the species to establish elsewhere.

It is interesting that a CMI threshold of <0.7 is necessary in all of our RMC analyses to include known location records. With only three factors (minimum and maximum temperature and soil moisture) used in the analysis, it should be easier to achieve a high CMI than with the four default factors (minimum and maximum temperature, rainfall amount and pattern) commonly used in analyses where a CMI of 0.7 is considered to be the standard [42,44,71]. Although another study [70] used a different approach in an RMC analyses, these authors also only use three factors (minimum and maximum temperature and rainfall—they do not specify whether they used rainfall amount or rainfall pattern, although rainfall amount would have been the more plausible parameter to have used), yet still conclude that a CMI > 0.7 is a good threshold to use in pest risk analyses. This appears to be the first RMC analysis attempting to compare the climate of known invaded regions with that of known native locations. Other RMC analyses pool all (or most) known location records as the “home” locations to be compared to the rest of the world, to identify regions at risk of a pest. Clearly, as these analyses have shown, the climates of both native and exotic locations of *T. mitsukurii* are not very similar, no doubt because climate in the native distribution does not experience the range of climates found in Italy and Australia. In an RMC analysis, by definition, all input (home) locations have a perfect match with themselves; hence, had we used both native and exotic location records as “home” locations to compare to the rest of the world, all known location records would necessarily have a high CMI.

There may be a number of reasons that the climate of the native distribution of *T. mitsukurii* does not closely match that of its exotic distribution. Firstly, there is only a small pool (32 locations) of known native location records that do not appear to capture the full extent of the climatic range of *T. mitsukurii* in Asia. Considering the projection of the exotic locations back into the native range, there is an absence of records from Yunnan Provence (Figure A5a), where the match index is quite high. There are locality records for *T. japonicus* (and *H. halys*) from that region, and *T. mitsukurii* and *T. japonicus* coexist elsewhere in China. Hence, it is unlikely that competitive exclusion explains this anomaly. The most plausible explanation, therefore, is that this is simply a case of a lack of observations being reported from this region. Given the widespread nature of the reported native range, it is unlikely that geographical barriers could explain the lack of records in these locations with warm, summer-dominant rainfall. While there may be a greater degree of interspecific competition in these parts of Asia that restricts distribution, the presence of *T. japonicus* in Yunnan suggests that this may not be the case.

In the RMC analyses where both Asian and Australian locations are treated as native, the analysis using soil moisture (Figure A6a) includes all areas shown to be a match to Asian locations (Figure A3a), which is to be expected. Many of the more northerly areas of Europe, North America and China that show a high match in Figure A5a do not have a high match in Figure A6a. Very little of Europe has a climate similar to that in Asia (Figure A3a), and because we are not including any Italian records as “home” locations in this run, there is not the same level of match throughout the rest of Europe, North America and northern China as in Figure A5a. Although a separate figure is not provided to show this, one of these Italian location records is excluded with a CMI of 0.7 in Figure A6a. This is somewhat problematic, given that *T. mitsukurii* occurs and is spreading in Italy [17,27,48,79], implying that the climate in Italy is suitable. This would suggest that the location records available in both Australia and Asia do not reflect the full climatic tolerances of *T. mitsukurii*. By contrast, the RMC using the four factors of minimum temperature, maximum temperature, rainfall amount and rainfall pattern (Figure A6b) includes all location records for *T. mitsukurii*, and projects more areas globally to have climates similar to where this species is currently found. Moreover, not surprisingly because the Australian location records are included in this analysis, an overall larger area globally is projected to match the climates of the Australian and Asian records (Figure A6b) than in the comparison to the Asian records only (Figure A3c).

These analyses highlight the sensitivity of a climate matching analysis to the completeness of the training locations when using a correlative model to assess the potential distribution of a species. Climate matching is generally considered to be a simplistic approach [31,43], as it only allows the user to compare the climates of different places, with no reference to laws underpinning a species response to the climate: Law of the Minimum [88] and Law of Tolerance [89]. Our analyses support this shortcoming, by demonstrating that the climate in the native range (based on known distribution records) is not very similar to that in the invaded range. This applies regardless of whether we consider *T. mitsukurii* to be native only to Asia, or to both Asia and Australia. Whilst there may be identifiable reasons for this mismatch, it nonetheless poses issues with using a climate-matching package to identify the potential range of a species, as has long been known [42,45,80]. This result reinforces the view that native range-based climate matching analyses should be considered cautiously and treated as likely conservative.
